# Impacts of ocean warming and acidification on the energy budget of three commercially important fish species

**DOI:** 10.1093/conphys/coac048

**Published:** 2022-07-21

**Authors:** José M Moreira, Ana Candeias Mendes, Ana Luísa Maulvault, António Marques, Rui Rosa, Pedro Pousão-Ferreira, Tânia Sousa, Patrícia Anacleto, Gonçalo M Marques

**Affiliations:** MARETEC—Marine, Environment & Technology Center, LARSyS, Instituto Superior Técnico, University of Lisbon, Av. Rovisco Pais 1, 1049-001 Lisboa, Portugal; Division of Aquaculture, Upgrading and Bioprospection (DivAV), Portuguese Institute for the Sea and Atmosphere (IPMA, I.P.), Av. Doutor Alfredo Magalhães Ramalho 6, 1495-165 Lisboa, Portugal; Division of Aquaculture, Upgrading and Bioprospection (DivAV), Portuguese Institute for the Sea and Atmosphere (IPMA, I.P.), Av. Doutor Alfredo Magalhães Ramalho 6, 1495-165 Lisboa, Portugal; MARE—Marine and Environmental Sciences Centre, Guia Marine Laboratory, Faculty of Sciences, University of Lisbon, Av. Nossa Sra do Cabo 939, 2750-374 Cascais, Portugal; UCIBIO-REQUIMTE, Applied Molecular Biosciences Unit, Department of Chemistry, NOVA School of Science and Technology—NOVA University of Lisbon, Campus de Caparica, 2829-516 Caparica, Portugal; Division of Aquaculture, Upgrading and Bioprospection (DivAV), Portuguese Institute for the Sea and Atmosphere (IPMA, I.P.), Av. Doutor Alfredo Magalhães Ramalho 6, 1495-165 Lisboa, Portugal; Interdisciplinary Centre of Marine and Environmental Research (CIIMAR), University of Porto, Terminal de Cruzeiros do Porto de Leixões, Av. General Norton de Matos, S/N, 4450-208, Matosinhos, Portugal; MARE—Marine and Environmental Sciences Centre, Guia Marine Laboratory, Faculty of Sciences, University of Lisbon, Av. Nossa Sra do Cabo 939, 2750-374 Cascais, Portugal; Department of Animal Biology, Faculty of Sciences, University of Lisbon, Campo Grande, 1749-016 Lisboa, Portugal; Division of Aquaculture, Upgrading and Bioprospection (DivAV), Portuguese Institute for the Sea and Atmosphere (IPMA, I.P.), Av. Doutor Alfredo Magalhães Ramalho 6, 1495-165 Lisboa, Portugal; MARETEC—Marine, Environment & Technology Center, LARSyS, Instituto Superior Técnico, University of Lisbon, Av. Rovisco Pais 1, 1049-001 Lisboa, Portugal; Division of Aquaculture, Upgrading and Bioprospection (DivAV), Portuguese Institute for the Sea and Atmosphere (IPMA, I.P.), Av. Doutor Alfredo Magalhães Ramalho 6, 1495-165 Lisboa, Portugal; MARE—Marine and Environmental Sciences Centre, Guia Marine Laboratory, Faculty of Sciences, University of Lisbon, Av. Nossa Sra do Cabo 939, 2750-374 Cascais, Portugal; Interdisciplinary Centre of Marine and Environmental Research (CIIMAR), University of Porto, Terminal de Cruzeiros do Porto de Leixões, Av. General Norton de Matos, S/N, 4450-208, Matosinhos, Portugal; MARETEC—Marine, Environment & Technology Center, LARSyS, Instituto Superior Técnico, University of Lisbon, Av. Rovisco Pais 1, 1049-001 Lisboa, Portugal

**Keywords:** ocean warming, ocean acidification, fish metabolism, Dynamic Energy Budget, climate change

## Abstract

A mechanistic model based on Dynamic Energy Budget (DEB) theory was developed to predict the combined effects of ocean warming, acidification and decreased food availability on growth and reproduction of three commercially important marine fish species: white seabream (*Diplodus sargus*), zebra seabream (*Diplodus cervinus*) and Senegalese sole (*Solea senegalensis*). Model simulations used a parameter set for each species, estimated by the Add-my-Pet method using data from laboratory experiments complemented with bibliographic sources. An acidification stress factor was added as a modifier of the somatic maintenance costs and estimated for each species to quantify the effect of a decrease in pH from 8.0 to 7.4 (white seabream) or 7.7 (zebra seabream and Senegalese sole). The model was used to project total length of individuals along their usual lifespan and number of eggs produced by an adult individual within one year, under different climate change scenarios for the end of the 21st century. For the Intergovernmental Panel on Climate Change SSP5–8.5, ocean warming led to higher growth rates during the first years of development, as well as an increase of 32–34% in egg production, for the three species. Ocean acidification contributed to reduced growth for white seabream and Senegalese sole and a small increase for zebra seabream, as well as a decrease in egg production of 48–52% and 14–33% for white seabream and Senegalese sole, respectively, and an increase of 4–5% for zebra seabream. The combined effect of ocean warming and acidification is strongly dependent on the decrease of food availability, which leads to significant reduction in growth and egg production. This is the first study to assess the combined effects of ocean warming and acidification using DEB models on fish, therefore, further research is needed for a better understanding of these climate change-related effects among different taxonomic groups and species.

## Teaser

Using experimental data of three commercially important marine fish species (*Diplodus sargus*, *Diplodus cervinus* and *Solea senegalensis*), a model based on Dynamic Energy Budget theory was parametrized. The model was used to produce projections of growth and reproduction for these species, under different scenarios of ocean warming and acidification.

## Introduction

Emissions of greenhouse gases (GHGs) have been increasing since the industrial revolution, with the atmospheric concentrations of CO_2_, CH_4_ and N_2_O now reaching unprecedented levels in the past 800 000 years (IPCC, 2021). This increase is attributed to combustion of fossil fuels for human activities. Increase in GHGs emissions strengthens the greenhouse effect causing global warming, including the warming of the oceans. Depending on the shared socio-economic pathway (SSP) scenario, the Intergovernmental Panel on Climate Change (IPCC), on their sixth assessment report (AR6), projects sea surface temperature (SST) to increase from 0.86°C (SSP1–2.6) to 2.89°C (SSP5–8.5) until the end of the 21st century. About 25% of emitted CO_2_ has been absorbed by the oceans (Sabine *et al.,* 2004), where it reacts with water to form carbonic acid, lowering pH. IPCC’s AR6 projects a decrease in global ocean surface pH from 0.05 (SSP1–2.6) to 0.4 units (SSP5–8.5) until the end of the 21st century.

Metabolic rates, such as growth, reproduction and maintenance, increase with the internal temperature of an organism—hence, for ectothermic animals, also with environmental temperature. The increase in metabolic rates is well described by the Arrhenius equation (Logan, 1982), which is supported by empirical evidence that the logarithm of metabolic rates decreases linearly with the inverse of absolute body temperature, on the cold side of the optimum temperature. As a result, growth rates of fish are generally higher at higher temperatures (Table 1), but only until a certain optimal temperature for growth, after which growth rates start declining. This optimal temperature is usually higher than the temperature that these fish experience in their natural environment, an indication that there must be a balancing of several factors such as food conversion efficiency or resistance to starvation (Handeland *et al.,* 2008). Optimal temperature can also change with age, with younger fish usually preferring warmer temperatures (McCauley and Huggins, 1979).

**Table 1:** Literature review of temperature and pH effects on growth and reproduction of fish species

External factor	Metabolic process	Species	Empirical evidence	Source
Temperature	Growth	*Solea senegalensis*	24–28% increase in the specific growth rate of larvae at 22°C, compared with 18°C	Pimentel *et al.* (2014)
		*Pleuronectes platessa, Platichtys flesus*	Growth rate increased with temperature, but declined at temperatures higher than 22°C	Fonds *et al.* (1992)
	Reproduction	*C. lumpus*	Sexually matured individuals at 14°C had a 50% reduction in sperm density, compared with pre-treatment levels (ambient temperature from 5 to 13°C)	Pountney *et al.* (2020)
		*A. melanopus*	78–87% decline in the number of egg clutches laid by individuals at 31.5°C, compared with 28.5°C	Miller *et al.* (2015)
pH	Growth	*A. melanopus*	Juveniles at 1032 μatm CO_2_ grew significantly less than juveniles at 430 μatm CO_2_	Miller *et al.* (2012)
		*T. albacares*	Larval length decreased by 20% at pH = 7.6, 12% at pH = 7.3 and 41% at pH = 6.9, compared to pH = 8.1	Frommel *et al.* (2016)
		*Amphiprion percula*	At pH = 7.8, larval length increased 15–18% and weight increased 47–52%, compared with pH = 8.1	Munday *et al.* (2009)
		*Paralichthys dentatus*	Larvae at pH = 7.1 developed earlier and were larger until the mid-larval period, compared with pH = 7.8	Chambers et al. (2014)
		*G. morhua*	Larvae were not significantly affected at *p*CO_2_ = 3200 μatm	Frommel et al. (2013)
		*Acanthochromis polyacanthus*	Elevated *p*CO_2_ had no effect on larvae growth (850 μatm)	Munday et al. (2011)
	Reproduction	*A. melanopus*	2-fold increase in the number of egg clutches and 67% more eggs per clutch at 1032 μatm CO_2_, compared with 430 μatm CO_2_	Miller et al. (2013)
		*Amphiprion percula*	45–75% increase in the number of egg clutches and 47–56% more eggs per clutch at 652–912 μatm CO_2_, compared with 446 μatm CO_2_	Welch and Munday (2016)
		*Acanthochromis polyacanthus*	Two-third decrease in the number of egg clutches at 912 μatm CO_2_, compared with 446 μatm CO_2_	Welch and Munday (2016)

Regarding ocean warming effects on reproduction, while higher temperatures have generally been shown to speed the gonadal development of fish (Sims *et al.,* 2004; Kjesbu *et al.,* 2010), which is consistent with an increase in reproduction rate, this rate can also decline if a certain temperature threshold is exceeded, leading to reports of decreased reproductive output at high temperatures (Table 1). However, it should be noted that if all individuals are fed with the same amount of food regardless of temperature treatment, the decrease in reproductive performance could be related to a higher energy demand from the individuals at higher temperatures.

Since metabolic rates increase with temperature, food requirements also increase. Up to a certain temperature threshold, if an organism ingests enough food, these requirements will be met. However, at high-enough temperatures, the food intake may not be enough to sustain the overall energy demand, leading to deprioritization of nonessential functions such as growth, reproduction or immunity. Moreover, prolonged exposure to high temperatures during the early life stages may change the ability to cope with additional stressors in the future (Rosa *et al.*, 2014; Alfonso *et al.,* 2021). This is of particular concern when considering climate change, since global warming is associated with other environmental problems—e.g. low pH, eutrophication, changes in ocean circulation—which induce stressful conditions like ocean acidification, hypoxia and salinity changes (IPCC, 2021; Sampaio *et al.,* 2021).

Many lifeforms in marine ecosystems suffer with ocean acidification. As CO_2_ is absorbed, the saturation state of aragonite and calcite lowers, hindering the formation of these minerals and facilitating their dissolution. That leads to slower calcifying rates and the weakening of the structures of calcifying organisms, which become more prone to erosion damage (Guinotte and Fabry, 2008; Pimentel *et al.,* 2014). Corals, echinoderms and mollusks, for example, are becoming increasingly more vulnerable, which results in less food availability for fish and deterioration of coral reefs that serve as their habitat. Even though fish are not considered calcifying organisms, they also produce CaCO_3_ precipitates in their inner ear, where they form concretions named otoliths, and in their intestinal lumen, where they contribute to water absorption and osmoregulation. Presumably due to effects on the formation of these precipitates, loss of hearing and olfactory function has been found on fish under acidification conditions, affecting their ability to find food (Cripps *et al.,* 2011). Heuer and Grosell (2014) hypothesize that these acidification effects are not related to precipitate formation, but to neurological disruption. On this subject, Pimentel *et al.* (2016) reported reduced capture rates of prey for gilthead seabream (*Sparus aurata*) and meagre (*Argyrosomus regius*) larvae exposed to acidification conditions (pH = 7.5), when compared with larvae exposed to normal conditions (pH = 8.0).

Regarding ocean acidification effects on growth, results have been remarkably variable, with most studies focusing on early life stages. Studies have found both increased and decreased growth, as well as no effect on growth, for several different fish species. Studies on effects of acidification on reproduction have also provided opposite results (Table 1).

Dynamic Energy Budget (DEB) theory is a non-species-specific theory that describes and quantifies metabolic processes at the organism level (Kooijman, 2010). It consists of a framework that provides tools to quantify the metabolic rates of an organism as a function of temperature, food density and environmental stressors, allowing to predict the consequences of different aspects of environmental change from a metabolic perspective. Thus, DEB models are a useful tool to assess the effects of different temperature, food and pH conditions on fish.

Several studies have resorted to DEB models to assess or predict the impacts of climate change on fish populations. Most studies focus on effects of ocean warming since temperature is known to be a crucial abiotic factor affecting physiological processes, and temperature data is usually widely available and easily integrated in DEB models through the Arrhenius equation. Teal *et al.* (2012) investigated the growth of plaice (*Pleuronectes platessa*) and sole (*Solea solea*) in the North Sea in two separate years. Results obtained with DEB models using data on stomachal content and seawater temperature were consistent with the observed distributional shifts in that area between those years. Stavrakidis-Zachou *et al.* (2021) used a DEB model to predict the time needed for European seabass (*Dicentrarchus labrax*) and meagre (*A. regius*) to reach their common market size in several sites in Greece, using SST projections. Pecquerie *et al.* (2009) used a biogeochemical model coupled with a hydrodynamic model to obtain data on seawater temperature and zooplankton biomass, which was then used as input on a DEB model to make predictions of growth and spawning location, timing, duration and performance of European anchovy (*Engraulis encrasicolus*) on the Bay of Biscay. Using a similar combination of models, Gatti *et al.* (2017) obtained predictions that were used to explain differences in the population dynamics of European anchovy and sardine (*Sardina pilchardus*) in the Bay of Biscay, revealing different strategies regarding energy acquisition and allocation to spawning.

While temperature effects on metabolism are already universally included on the DEB framework with the Arrhenius equation, the acidification effects are not. To date, there is a limited number of studies applying DEB theory on ocean acidification effects and, to the best of our knowledge, none of them concerning fish species. Most of these studies try to include acidification effects on a DEB model by varying specific parameters to fit experimental data under different pH or *p*CO_2_ conditions. Muller and Nisbet (2014) studied the impact of acidification on growth and calcifying rates on a species of coccolithophore (*Emiliania huxleyi*) by varying the specific assimilation and maintenance rates, and energy conductance, as a function of H^+^ concentration beyond a no-effect concentration threshold. Jager *et al.* (2016) studied the impact of ocean acidification on green sea urchins (*Strongylocentrotus droebachiensis*), considering that acidification acted as a stress factor on assimilation, maintenance and growth. The stress factors were estimated by fitting the model output with experimental data at different pH values. Klok *et al.* (2014) changed the assimilation, maintenance and growth rates by directly varying, with pH, the parameters assimilation efficiency, volume-specific somatic maintenance cost and volume-specific cost of structure, to fit experimental data of cockles (*Cerastoderma edule*). Similarly, Ren *et al.* (2020) used data from laboratory experiments to directly estimate the values of the same three parameters for green-lipped mussel (*Perna canaliculus*) at different pH levels. Finally, Maynou *et al.* (2020) modified the assimilation and maintenance rates, but not the growth rate, by varying the ingestion rate and the volume-specific somatic maintenance rate of a clam species (*Ruditapes philippinarum*), to estimate the parameters with different temperature and pH values.

In our study, a mechanistic model based on DEB theory is applied to three commercially important marine fish species: white seabream (*Diplodus sargus*), zebra seabream (*Diplodus cervinus*) and Senegalese sole (*Solea senegalensis*). The model aims to predict the combined impact of pH and temperature on metabolism. Different combinations of temperature and pH were tested, along with different levels of food availability, since ocean warming and acidification are linked with a decrease in the net primary productivity of marine environments (IPCC, 2019). This resulted in projections of growth and reproduction for a wide range of possible climate change scenarios.

We model the pH effect by modifying the volume-specific somatic maintenance cost parameter to fit experimental data of fish exposed to acidification. This choice resulted from the available empirical data, which consists of length and weight measurements through time, prompting the variation of a parameter related with metabolic processes that affect growth. These are, in DEB theory, the assimilation, growth and somatic maintenance processes. However, on DEB ecotoxicology studies, growth is a very uncommon physical mode of action when compared with assimilation and maintenance, which are the two most common (Ashauer and Jager, 2018). The volume-specific somatic maintenance cost, $[{\dot{p}}_M]$, the main parameter that defines the somatic maintenance process, was thus chosen as the parameter to be affected by acidification on the model used in this work, but this assumption does not discard an effect on assimilation. In fact, by varying the functional response ($f$), a variable representative of food availability, in the projections of this model, a simultaneous effect in assimilation was tested, since an effect in the surface-area-specific maximum assimilation rate $\{{\dot{p}}_{Am}\}$ is indistinguishable from an effect in $f$, as they are both only used together to define assimilation.

**Figure 1:**
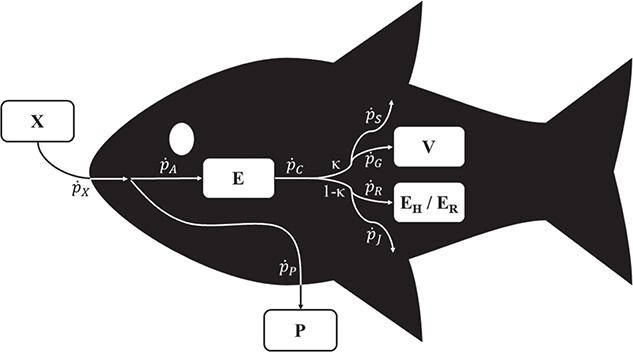
Diagram of a DEB model. $X$, food; $P$, feces; $E$, reserve; $V$, structure; ${E}_H$, maturity; ${E}_R$, reproduction buffer; ${\dot{p}}_X$, ingestion flux; ${\dot{p}}_P$, defecation flux; ${\dot{p}}_A$, assimilation flux; ${\dot{p}}_C$, mobilization flux; ${\dot{p}}_S$, somatic maintenance flux; ${\dot{p}}_G$, growth flux; ${\dot{p}}_R$, reproduction flux; ${\dot{p}}_J$, maturity maintenance flux; $\kappa$, fraction of mobilized flux allocated to soma. The mathematical description of these variables and fluxes are presented in Table 2.

## Methodology

### DEB model

DEB theory provides a mathematical foundation to quantify the metabolism of any living organism at the individual level, built upon physicochemical principles and thermodynamic laws such as conservation of mass and energy. It allows for a mechanistic approach to study the different processes involved in metabolism, such as feeding, maintenance, development, growth and reproduction, across the whole life cycle from fecundation to death, while accounting for effects of environmental conditions (usually temperature and food availability) (Sousa *et al.,* 2008; Kooijman, 2010; Meer *et al.,* 2014).

An organism described by a DEB model is fully described by four state variables: reserve ($E$), structure ($V$), maturity (${E}_H$) and reproduction buffer (${E}_R$). Reserve is the energy accumulated from the assimilation of ingested food. Energy from the reserve is then mobilized and used for different metabolic functions including growth, maintenance, maturation and reproduction. Structure, which is biomass attained by growth, controls the structural size of the organism and the assimilation process that is proportional to structural surface area. Maturity amounts to the energy invested to complexify the organism and prepare it for life stage transitions. The reproduction buffer is the energy stored for reproduction.

Energy flowing within an organism for different metabolic purposes can be accounted through a series of energy rates or fluxes. These are defined as a function of state variables and a set of parameters (Fig. 1; Table 2).

**Table 2:** General equations of a DEB model

State variables		Metabolic rates	
Reserve	$\frac{dE}{dt}={\dot{p}}_A-{\dot{p}}_C$	Ingestion	${\dot{p}}_X={\dot{p}}_A/{\kappa}_X$
Structure	$\frac{dV}{dt}=\frac{{\dot{p}}_G}{[{E}_G]}$	Defecation	${\dot{p}}_P={\kappa}_P\ {\dot{p}}_X$
Maturity	$\frac{d{E}_H}{dt}=\begin{cases}{\dot{p}}_R\quad \mathrm{if}\ {E}_H<{E}_H^p\\ {}0\quad\ \, \, \mathrm{otherwise}\end{cases}$	Assimilation	${\dot{p}}_A=\begin{cases}0\qquad\qquad\quad\ \ \mathrm{if}\ {E}_H<{E}_H^b\\ {}\{{\dot{p}}_{Am}\}\ f\ {V}^{2/3}\ \, \, \mathrm{otherwise}\end{cases}$
Reproduction buffer	$\frac{d{E}_R}{dt}=\begin{cases}0\qquad\, \mathrm{if}\ {E}_H<{E}_H^p\\ {}{\kappa}_R\ {\dot{p}}_R\ \mathrm{otherwise}\end{cases}$	Mobilization	${\dot{p}}_C=E\frac{[{E}_G]\dot{v}/{V}^{1/3}+[{\dot{p}}_S]}{\kappa [E]+[{E}_G]}$
		Somatic maintenance	${\dot{p}}_S=[{\dot{p}}_M]\ V$
		Growth	${\dot{p}}_G=\kappa\ {\dot{p}}_C-{\dot{p}}_S$
		Maturity maintenance	${\dot{p}}_J={\dot{k}}_J\ {E}_H$
		Reproduction	${\dot{p}}_R=(1-\kappa )\ {\dot{p}}_C-{\dot{p}}_J$

A full description of the parameters in the equations is presented in Table 3.

Several typified DEB models (Marques *et al.,* 2018) have been developed to describe the growth of specific organisms. The most commonly used typified model for bony fish species, and the one used in this study, is the DEB *abj* model (AmP, 2021). In this model, an organism goes through four life stages: embryo, juvenile I, juvenile II and adult. These are separated by three critical transitions: birth separates the embryo and juvenile I stages, metamorphosis separates the juvenile I and juvenile II stages and puberty separates the juvenile II and adult stages. These transitions occur when maturity reaches specific thresholds, simply denominated as maturity at birth (${E}_H^b$), maturity at metamorphosis (${E}_H^j$) and maturity at puberty (${E}_H^p$). For estimation purposes, we have also included hatching as the moment where the organism gets out of the egg, as birth is defined as the moment when the organism starts feeding. In the same way, hatching occurs when the maturity at hatching threshold (${E}_H^h$) has been reached.

During the embryo stage, the organism does not ingest food and does not reproduce; during the juvenile I and II stages, the organism ingests food but does not reproduce; and during the adult stage, the organism ingests food and reproduces. The juvenile I stage differs from juvenile II by presenting accelerated growth. In this case, the parameters surface-area-specific maximum assimilation rate and energy conductance increase as a function of length, between birth and metamorphosis:

$$ \left\{{\dot{p}}_{Am}\right\}={\left\{{\dot{p}}_{Am}\right\}}_b\ \frac{L}{L_b} $$

and

$$ \dot{v}={\dot{v}}_b\ \frac{L}{L_b}, $$

where ${L}_b$ is the length at birth and ${\{{\dot{p}}_{Am}\}}_b$ and ${\dot{v}}_b$ are the values of these parameters until birth. After metamorphosis, these two parameters are constant again:

$$ \left\{{\dot{p}}_{Am}\right\}={\left\{{\dot{p}}_{Am}\right\}}_b\ \frac{L_j}{L_b} $$

and

$$ \dot{v}={\dot{v}}_b\ \frac{L_j}{L_b}, $$

where ${L}_j$ is the length at metamorphosis.

After metamorphosis, with constant maximum assimilation rate and energy conductance, if the functional response ($f$) is constant, growth in the juvenile II and adult stages is described by the von Bertalanffy equation:

$$ L(t)={L}_{\infty }-\left({L}_{\infty }-{L}_b\right)\ \exp \left(-t\ {\dot{r}}_B\right) $$

where $L$ is length, ${L}_b$ is the length at birth, ${L}_{\infty }$ is ultimate length, $t$ is time since birth and ${\dot{r}}_B$ is the von Bertalanffy growth rate.

**Table 3:** Description of DEB parameters

Parameter	Symbol	Units	Description
Arrhenius temperature	${T}_A$	K	Sensitivity to temperature change
Zoom factor	$z$	-	Ratio of maximum length ${L}_m=\frac{\kappa\ \{{\dot{p}}_{Am}\}}{[{\dot{p}}_M]}$ and the maximum length of a reference species with ${L}_m^{ref}=1$ cm
Specific search rate	$\{{\dot{F}}_m\}$	d^−1^ cm^−2^	Organism’s ability to search for food
Digestion efficiency	${\kappa}_X$	-	Efficiency of food transformation into reserve
Defecation efficiency	${\kappa}_P$	-	Efficiency of food transformation into feces
Energy conductance	$\dot{v}$	cm d^−1^	Energy velocity
Fraction of mobilized flux allocated to soma	$\kappa$	-	Fraction of mobilized energy allocated to the somatic maintenance and growth processes
Reproduction efficiency	${\kappa}_R$	-	Fraction of reproduction energy that is stored and later released at spawning
Volume-specific somatic maintenance cost	$[{\dot{p}}_M]$	J d^−1^ cm^−3^	Somatic maintenance costs by unit of volume
Maturity maintenance rate coefficient	${\dot{k}}_J$	d^−1^	Ratio of maturity maintenance rate and cumulative energy invested into maturation
Volume-specific cost of structure	$[{E}_G]$	J cm^−3^	Transformation cost of reserve into structure by volume
Maturity at hatching	${E}_H^h$	J	Cumulative energy invested into maturation until hatching
Maturity at birth	${E}_H^b$	J	Cumulative energy invested into maturation until birth
Maturity at metamorphosis	${E}_H^j$	J	Cumulative energy invested into maturation until metamorphosis
Maturity at puberty	${E}_H^p$	J	Cumulative energy invested into maturation until puberty
Weibull aging acceleration	${\ddot{h}}_a$	d^−2^	Regulates the organism’s lifespan
Gompertz stress coefficient	${s}_G$	-	Regulates the organism’s lifespan
Shape coefficient	${\delta}_M$	-	Ratio of structural length and physical length
Acidification stress factor	$s$	-	Change in somatic maintenance costs due to acidification stress

To quantify the acidification effect, the volume-specific somatic maintenance cost, $[{\dot{p}}_M]$, was modified by adding a stress factor ($s$), as follows:

$$ \left[{\dot{p}}_M\right]=\left(1+s\right)\ {\left[{\dot{p}}_M\right]}_0, $$

where $[{\dot{p}}_M]$ is the parameter’s corrected value and ${[{\dot{p}}_M]}_0$ is the base value in no-stress conditions.

Contrarily to the work by Muller and Nisbet (2014), for example, where the chemistry of the calcifying process of coccolithophores was taken into account in a DEB model, here we account and quantify the acidification effects without detailing the underlying processes causing them.

A summary of the parameters used in the model of this work, along with their name and description, is presented in Table 3.

### Experimental data

Experimental data from two different sources, together with data from bibliographical references, were used to estimate a set of parameters that define the DEB model for each species.

Data from the Olhão Pilot Fish Farming Station of the Portuguese Institute for Sea and Atmosphere (EPPO-IPMA) included measurements of total length and wet weight through time, for the three studied species. The measured individuals were as follows: white seabream juveniles between 77 and 377 days post-hatching (dph), zebra seabream juveniles between 129 and 179 dph and Senegalese sole adults between 2 and 7 years. All individuals were maintained at temperature and pH conditions similar to the outside environment of Ria Formosa, a coastal lagoon on the Portuguese south coast.

Data from Guia Marine Laboratory (MARE-FCUL) included the same type of measurements for the same three species, but under different environmental conditions. Fish were exposed to four combinations of constant temperature and pH. Temperature conditions were based on IPCC projections of global surface temperature, corresponding to the SSP5–8.5 scenario, which predicts an increase between 3.3 and 5.7°C until the end of the 21st century. With a baseline temperature of 19°C, the experiments with white seabream (the first preliminary trial, with a worst-case scenario) simulated ΔT = +5°C, while the experiments with zebra seabream and Senegalese sole simulated ΔT = +4°C. The pH conditions used in the experiments were also based on the IPCC projections with a decline corresponding approximately to the SSP5–8.5 scenario. A decrease in pH levels of 0.3 units was chosen for the zebra seabream and Senegalese sole experiments based on the prediction for the end of the 21st century, while a decrease of 0.6 units was chosen for the white seabream experiment, based on a more extreme prediction for the end of the 23rd century. Fish were firstly acclimated to laboratory conditions for 1 month and then the temperature was slowly increased (1°C per day) and pH was slowly lowered (−0.1 pH units per day) until reaching the conditions of climate change previously described. All individuals were juveniles: white seabream started from 147 dph and aged to 224 dph, zebra seabream from 154 to 232 dph and Senegalese sole from 298 to 373 dph. Fish specimens were fed with 2–3% of their average body weight (divided into two portions per day) throughout the experimental period, with fish diets being manufactured by Sparos, Lda (Olhão, Portugal) considering the respective nutritional requirements of each species.

The graphs of total length and wet weight through time, in the EPPO-IPMA and MARE-FCUL experiments, are included in Appendix 2.

### Parameter estimation

The parameter estimation was conducted according to the AmP method (AmP is the acronym for Add-my-Pet which is an online open-access project that collects individual metabolic data and estimates DEB parameters). The files from the AmP collection (AmP, 2021) on the three studied species were modified to include the experimental data, along with data from other references.

The AmP method consists in estimating DEB parameters by minimizing the difference between collected data and a set of predictions produced by the DEB model, through a determined loss function and using the Nelder–Mead numerical method. The loss function used for the parameter estimation was the symmetric bounded loss function described in Marques *et al.* (2019).

The data used in the estimation procedure included zero-variate data, i.e. datasets comprising a single point (e.g. age, length and weight at specific life events such as birth, metamorphosis and puberty), and univariate data, i.e. datasets with multiple points (e.g. length and weight measurements through time). The EPPO-IPMA and MARE-FCUL experiments provided univariate data to the parameter estimation, while various references provided both zero-variate and univariate data. The full list of zero-variate and univariate data from all sources is included in Appendix 1.

### Projections

The estimated sets of parameters were used to define an *abj* DEB model in a simulation program developed on Matlab. Functional response ($f$) of the Holling type II was used as a forcing variable to assess how food availability may affect the results. (Forcing variable is a variable whose dynamics is controlled by factors that are external to the organism such as food density, environmental temperature, pH.)

The observable variable projected for growth predictions was total length, computed using the standard equations on the AmP method, along the expected lifespan of each species: 10, 11 and 17 years for the white seabream (FishBase, 2021a), zebra seabream (Pajuelo *et al.,* 2003b) and Senegalese sole (Teixeira and Cabral, 2010), respectively. For reproduction predictions, the projected observable variable was the number of eggs produced by an adult individual at its ultimate size, given by the energy invested into reproduction that was effectively converted to eggs, divided by the energy content of one egg (i.e. the organism’s initial reserve), on a period of 1 year:

$$ {\displaystyle \begin{array}{c}{n}_{\mathrm{eggs}}=\frac{\kappa_R\ {\dot{p}}_R}{E_0}\Delta t,\end{array}} $$

where ${\kappa}_R$ is the reproduction efficiency, ${\dot{p}}_R$ is the reproduction energy rate, ${E}_0$ is the energy content of the egg and $\Delta t$ equals 365 days.

The simulation program was tested with varying temperature corresponding to the typical seasonal variation in Ria Formosa, and with an average constant temperature throughout the year. There were no significant differences in the results, hence, for simplicity, final projections were made using an average constant temperature.

Because the IPCC projects an increase in sea surface temperature from 0.86°C (SSP1–2.6) to 2.89°C (SSP5–8.5) until the end of the 21st century (IPCC, 2021) the projections on this work include four temperature values: today’s yearly average temperature in Ria Formosa, 19°C, and increases of +1, +2 and +3°C. The projections will also assess four values of $f$: 1, 0.9, 0.8 and 0.7, to take into account that rises in temperature are often associated with a decline in food supply. The possible combinations of the four temperatures and the four $f$ values amounts to a total of 16 scenarios for no acidification conditions.

To make projections of the acidification effect an acidification stress factor ($s$) was included in the model. The acidification factor will not have a range of possible values, but rather only one of two values: zero, meaning no acidification stress at a pH of 8.0, or the value resulting from the parameter estimation, representing the effect on maintenance costs of a pH of 7.7 (zebra seabream and Senegalese sole) or 7.4 (white seabream). For this reason, for each species only two pH conditions were projected. Since the projections on the acidification effect in all the aforementioned conditions of temperature and food availability would double the number of scenarios to 32, which would hinder the comparison of the scenarios and the visibility of the graphs, only four combinations of temperature and functional response were tested. The four combinations simulated, based on the assumption of decreased food availability with increased temperature, were as follows: 19°C, $f$ = 1; 20°C, $f$ = 0.9; 21°C, $f$ = 0.8; and 22°C, $f$ = 0.7.

## Results and discussion

### Parameter estimation and validation

The estimated values for each parameter and each species are presented in Table 4. Adding to the parameters presented in Table 4, the functional response $f$ for the experimental data from MARE-FCUL and EPPO-IPMA was estimated as $f=1$ for all three species, suggesting *ad libitum* feeding conditions.

**Table 4:** Parameter estimation results for each species

Parameter	Symbol	Units	*D. sargus*	*D. cervinus*	*S. senegalensis*
Arrhenius temperature	${T}_A$	K	8000	8000	8000
Zoom factor	$\boldsymbol{z}$	**-**	**4.71**	**11.84**	**3.47**
Specific search rate	$\{{\dot{F}}_m\}$	d^−1^ cm^-2^	6.5	6.5	6.5
Digestion efficiency	${\kappa}_X$	-	0.8	0.8	0.8
Defecation efficiency	${\kappa}_P$	-	0.1	0.1	0.1
Energy conductance	$\dot{\boldsymbol{v}}$	**cm d** ^**-1**^	**0.04237**	**0.04468**	**0.05852**
Fraction of mobilized flux allocated to soma	$\boldsymbol{\kappa}$	**-**	**0.868**	**0.826**	**0.882**
Reproduction efficiency	${\kappa}_R$	-	0.95	0.95	0.95
Volume-specific somatic maintenance cost	$[{\dot{\boldsymbol{p}}}_{\boldsymbol{M}}]$	**J d** ^**−1**^ **cm**^**-3**^	**18.53**	**10.17**	**29.16**
Maturity maintenance rate coefficient	${\dot{k}}_J$	d^-1^	0.002	0.002	0.002
Volume-specific cost of structure	$[{\boldsymbol{E}}_{\boldsymbol{G}}]$	**J cm** ^**-3**^	**5231**	**5236**	**5226**
Maturity at hatching	${\boldsymbol{E}}_{\boldsymbol{H}}^{\boldsymbol{h}}$	**J**	**0.06622**	**-**	**0.01656**
Maturity at birth	${\boldsymbol{E}}_{\boldsymbol{H}}^{\boldsymbol{b}}$	**J**	**0.183**	**0.173**	**0.088**
Maturity at metamorphosis	${\boldsymbol{E}}_{\boldsymbol{H}}^{\boldsymbol{j}}$	**J**	**2.143**	**0.173**	**3.525**
Maturity at puberty	${\boldsymbol{E}}_{\boldsymbol{H}}^{\boldsymbol{p}}$	**J**	**7.06x10** ^**4**^	**5.54x10** ^**4**^	**7.59x10** ^**5**^
Weibull aging acceleration	${\ddot{\boldsymbol{h}}}_{\boldsymbol{a}}$	**d** ^**-2**^	**1.29x10** ^**–8**^	**6.26x10** ^**–9**^	**7.90x10** ^**–9**^
Gompertz stress coefficient	${s}_G$	-	0.0001	0.0001	0.0001
Shape coefficient	${\boldsymbol{\delta}}_{\boldsymbol{M}}$	**-**	**0.2241**	**0.2166**	**0.2218**
Acidification stress factor	$\boldsymbol{s}$	**-**	**0.3591**	**−0.0205**	**0.0564**

Parameters in boldface were estimated by minimizing the error between DEB model predictions and empirical data (AmP method). Parameters not in boldface were not estimated due to insufficient data and were set to the standard values in the AmP collection (AmP, 2021). A description of the parameters can be found in Table 3.

The estimated values for the acidification stress factor $s$ were, for the zebra seabream and the Senegalese sole, relatively close to zero, possibly being an indication of a small or practically null effect of acidification on these species. For the white seabream the estimated value of this parameter was much higher. A stronger acidification effect was already expected for this species since it was exposed to a lower pH on the MARE-FCUL experiments, but, given the much larger $s$ value when compared with the other two studied species, it is also possible that this species presents a higher vulnerability to ocean acidification. However, by varying only the $s$ value and maintaining the values of the other parameters, the relative error of the prediction of the data from MARE-FCUL (the only one accounting for pH, therefore the only affected by $s$) barely changed for the white seabream. For example, $s$ = 0 leads to predictions with similar goodness of fit of the MARE-FCUL data when compared to the estimated value $s$ = 0.3591. Therefore, even though this work projects growth and reproduction according to the latter, other values of $s$ could also be valid, along with their implications on the magnitude of the acidification effect on this species.

To make a preliminary validation of our estimated set of parameters for the three studied fish species, we compared them with the parameter values of six taxonomically proximate species and with all species estimated in the AmP collection (currently 3233) (AmP, 2021). The taxonomically proximate species selected were three species in the *Diplodus* genus: the sharpsnout seabream (*Diplodus puntazzo*), the annular seabream (*Diplodus annularis*) and the common two-banded seabream (*Diplodus vulgaris*); and three species of flatfish in the *Soleidae* family: the common sole (*S. solea*), the wedge sole (*Dicologlossa cuneata*) and the sand sole (*Pegusa lascaris*). We compared only parameters that are independent of the maximum size of the species (Sousa *et al.,* 2008; Kooijman, 2010) such as the somatic maintenance cost, the energy conductance and the fraction of mobilized flux allocated to soma. For more than 90% of all species in the collection, the energy conductance, the volume-specific somatic maintenance cost and the fraction of mobilized flow allocated to soma are respectively between 0.01 and 0.5 cm.day^−1^, 10 and 5000 J.cm^−3^.day^−1^ and 0.5 and 1. For the selected taxonomically proximate species the same parameters are respectively between 0.01 and 0.07 cm.day^−1^, 18 and 33 J.cm^−3^.day^−1^ and 0.75 and 1. For our three species, the energy conductance, the specific somatic maintenance cost and the fraction of mobilized flow allocated to soma vary respectively between 0.04 and 0.06 cm.day^−1^, 10–30 J.cm^−3^.day^−1^ and 0.82–0.88. These three parameters are within the set of values expected for taxonomically related species in the AmP collection when compared with the universe of other species already estimated in the collection.

**Figure 2:**
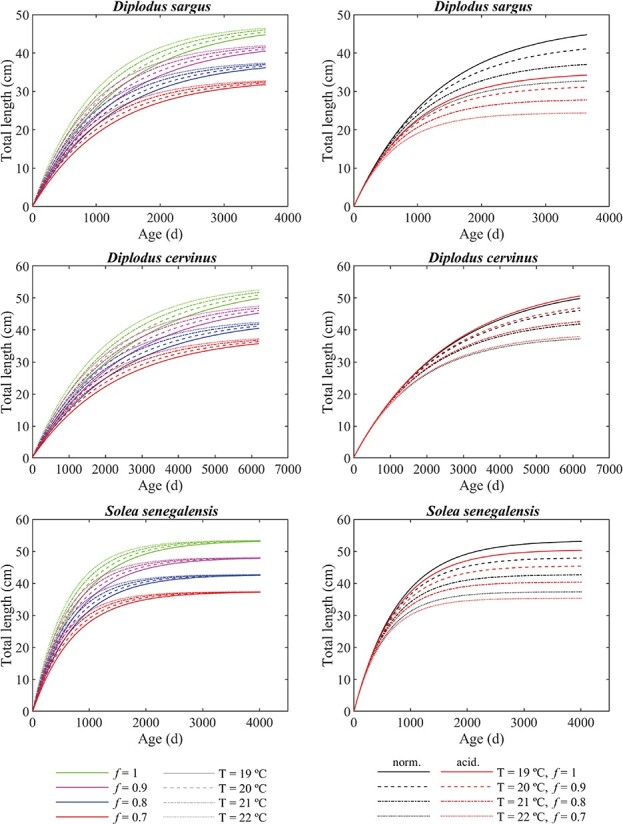
Projections of total length for the white seabream (top), zebra seabream (middle) and Senegalese sole (bottom), under different climate change scenarios. On the left, 16 scenarios with different combinations of temperature and food availability. On the right, 8 scenarios composed of four combinations of temperature and food availability, under normal pH conditions (norm.) (pH = 8.0) and under acidification conditions (acid.) (pH = 7.4 for the white seabream; pH = 7.7 for the zebra seabream and the Senegalese sole).

**Figure 3:**
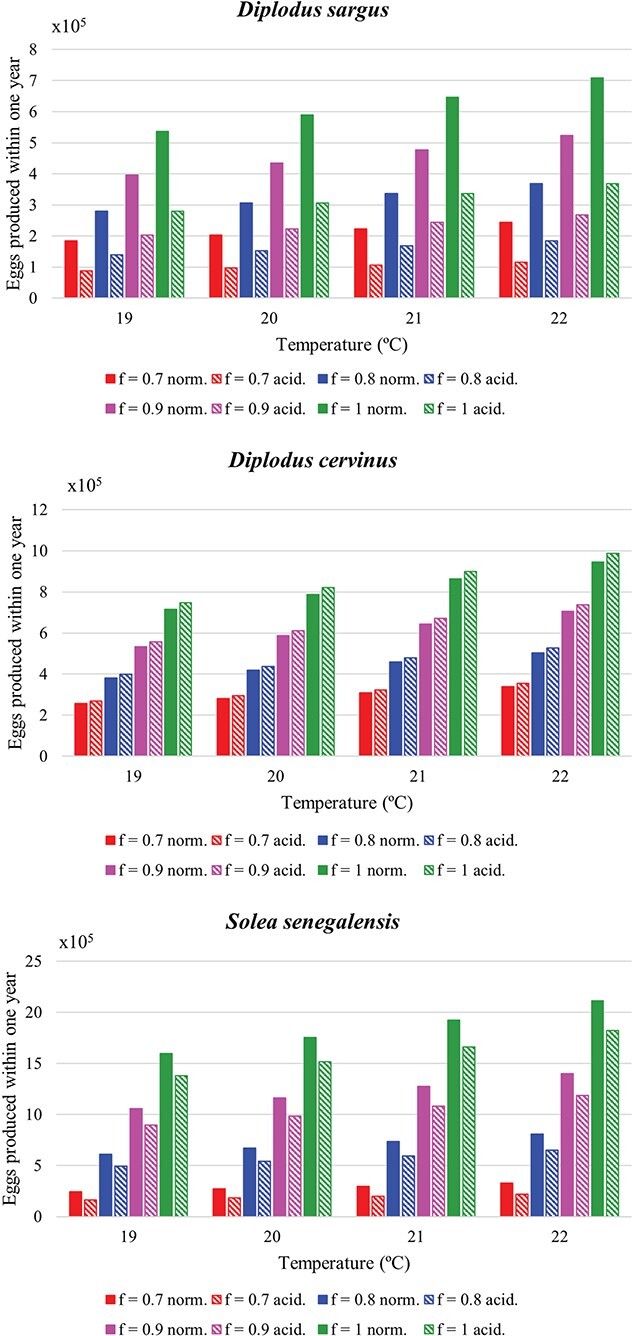
Projections of number of eggs produced within 1 year by an adult on its ultimate size, under different scenarios of temperature and food availability, under normal pH conditions (norm.) (pH = 8.0) and under acidification conditions (acid.) (pH = 7.4 for the white seabream; pH = 7.7 for the zebra seabream and the Senegalese sole)..

### Projections

Using the estimated values for the parameters on the simulation program resulted in the growth projections presented in Fig. 2.

All three studied species show higher growth rates at higher temperatures during their early development, up to about 3, 6 and 2 years of age, for white seabream, zebra seabream and Senegalese sole, respectively. After those periods, since the size of individuals at higher temperatures starts stabilizing sooner, growth rates become higher at lower temperatures. On the other hand, specific growth rates (SGRs), i.e. growth rates relative to body size, are higher at higher temperatures only up to the end of the juvenile I stage, where there is accelerated growth. Shortly after that, the SGR is higher at lower temperatures, as growth is higher relative to the smaller size of the individuals. Fonds *et al.* (1992) also observed a decrease in SGR with increasing temperature (18–22°C) for plaice (*P. platessa*), but not for flounder (*Platichthys flesus*). For Senegalese sole, one of the species in this work, Pimentel *et al.* (2014) observed a higher SGR at 22°C (6–7% per day) than at 18°C (5% per day), for individuals with an age of 30 dph. Nevertheless, for this species, our model shows close SGR values at that age, with ~4% per day for both 19°C (current temperature) and 22°C (SSP5–8.5), with only minimal difference between temperatures, regardless of functional response (within the 0.7–1.0 range). The differences between the results of the model and the observations in Pimentel *et al.* (2014) can be at least partially explained by the data used to parameterize the model being from considerably older individuals, and the assumption that ${T}_A$ is constant throughout the whole life cycle, i.e. temperature sensitivity is considered constant regardless of age. In the context of the IPCC projections for the end of the 21st century, our results show that, under the SSP5–8.5 scenario, temperature significantly accelerates the juvenile development of the three studied fish species, leading to consistently larger sizes throughout the whole life cycle, even though the size difference between this scenario and a current temperature scenario starts to decrease after a certain age.

A pH decrease of 0.3 units, corresponding to the SSP5–8.5 scenario projections for the end of the 21st century, resulted in consistently smaller sizes throughout the whole life cycle for Senegalese sole, compared with current pH conditions. Under the same conditions, the model showed slightly larger sizes for zebra seabream under decreased pH. A more accentuated decrease of 0.6 units, corresponding to a prediction for the end of the 23rd century under the same scenario, resulted in smaller sizes for white seabream, with the size difference being larger than for the Senegalese sole. Unlike with temperature changes, individuals will not reach the same ultimate size under different pH conditions if given enough time. In turn, projections showed consistently lower growth rates under acidification conditions for white seabream and Senegalese sole, while zebra seabream showed consistently higher growth rates, although very similar to normal pH conditions. The reduced growth for white seabream and Senegalese sole is in accordance with the observations of Miller *et al.* (2012) for cinnamon anemonefish (*Amphiprion melanopus*) and of Frommel *et al.* (2016) for yellowfin tuna (*Thunnus albacares*). On the other hand, growth projected for the zebra seabream under acidification resembles more an absence of effect as observed by Frommel *et al.* (2013) for Baltic cod (*Gadus morhua*) and by Franke and Clemmesen (2011) for Atlantic herring (*Clupea harengus*), rather than a meaningful increase in growth. For Senegalese sole, Pimentel *et al.* (2014) observed a lower SGR at pH = 7.5 (5–6% per day) than at pH = 8.0 (5–7% per day), for individuals with an age of 30 dph. In our model, a similar SGR value of 4% per day was observed for this species, at the same age, for pH = 7.7, regardless of temperature (range of 19–22°C) and of functional response (range of 0.7–1). In our case, the difference between the SGR at pH = 7.7 and pH = 8.0 is comparatively insignificant at this stage, only 3 × 10^−5^ percentual points.

Reproduction projections, for the initial 16 scenarios, in the form of number of eggs produced within 1 year by an adult on its maximum size, assuming no acidification effect and in the presence of an acidification effect, are presented in Fig. 3. Without surprise, egg production increased with temperature since the reproduction rate was set to increase in the model following the Arrhenius equation. Hence, at a temperature corresponding to the SSP5–8.5 scenario, it increased significantly compared to current temperature, between 32% and 34% for all three studied species. This contrasts with the results from Pountney *et al.* (2020) for lumpfish (*Cyclopterus lumpus*), and from Miller *et al.* (2015) for cinnamon anemonefish (*A. melanopus*), where other factors like food conversion efficiency may have favored lower temperatures, but is in accordance with the faster gonadal development of fish at higher temperatures in Sims *et al.* (2004) and Kjesbu *et al.* (2010).

Acidification had a significant effect on the number of eggs produced within 1 year by an adult white seabream, with a projected decrease between 48% and 52% for a pH of 7.4 when compared with a pH of 8.0. Under pH conditions of the SSP5–8.5 in the end of the 21st century (pH = 7.7), the Senegalese sole presented a decrease between 14% and 33%, while the zebra seabream presented an increase of 4–5%, when compared with the current pH of 8.0. The decline in egg production for white seabream and Senegalese sole is of similar magnitude as the 38% decrease in egg clutch production reported by Welch and Munday (2016) for spiny chromis. The projected increase in egg production for zebra seabream seems to represent a soft or negligible effect on reproductive output, contrarily to the 2-fold increase in egg clutch production and 67% increase in eggs per clutch for cinnamon anemonefish under acidification (1032 μatm CO_2_) in Miller *et al.* (2013), when compared with fish at control conditions (430 μatm CO_2_). The same can be said about the orange clownfish in Welch and Munday (2016), where increases of 45–75% in egg clutch production and of 47–56% in eggs per clutch were verified for fish exposed to acidification (652–912 μatm CO_2_) when compared with fish exposed to control conditions (446 μatm CO_2_).

The strong effect of the functional response on both growth and reproduction projections indicates that indirect effects of ocean warming and acidification, like depletion of food sources, habitat loss and loss of olfactory function leading to less food intake, may have a more negative impact than a direct effect through temperature increase and pH decrease. It should be mentioned that, by simulating four $f$ values, we are not only assessing the effect of different levels of food availability, but also possible effects on assimilation. In the second case, $f$ is acting as a proxy for the surface-area-specific maximum assimilation rate $\{{\dot{p}}_{Am}\}$, since an effect on one of these two parameters is indistinguishable from a similar effect on the other (note that they only appear in the form of a product in the assimilation rate equation; see Table 2).

## Concluding remarks

This is the first work studying the combined effects of ocean warming and acidification using a model based on DEB theory. As both phenomena occur in nature simultaneously, and are expected to increase in intensity, the insights provided by the presented results are particularly relevant to evaluate the consequences of environmental change on the three commercially important fish species studied in this work.

Future studies on the effects of ocean warming and acidification on marine life using DEB models can probably improve the current scientific knowledge by estimating parameters using more detailed experimental data. A wider timespan of *in vivo* experiments, during different stages of their life cycle and a wider range of pH levels are examples of how the data can improve the estimation of DEB parameters and thus the accuracy of the projections using this model. Because few studies have assessed the effects of ocean acidification using DEB models and, as far as the authors know, none of them concerning fish, further research is necessary for a better understanding of how these effects may vary between different organisms, and particularly between different fish species. Particular attention should be given to how different aspects of climate change—such as ocean warming and acidification—interact with each other and how they affect fish, both directly and indirectly through, for example, changes in food availability.

## Funding

This study was supported by the project FISHBUDGET - Effects of climate change on marine fish energy budgets (PTDC/BIA-BMA/28630/2017) from the Portuguese Foundation for Science and Technology (FCT).

FCT also supported the contract of Patrícia Anacleto in the framework of the CEECIND 2017 (CEECIND/01739/2017).

## Figures and Tables

**Figure 4:**
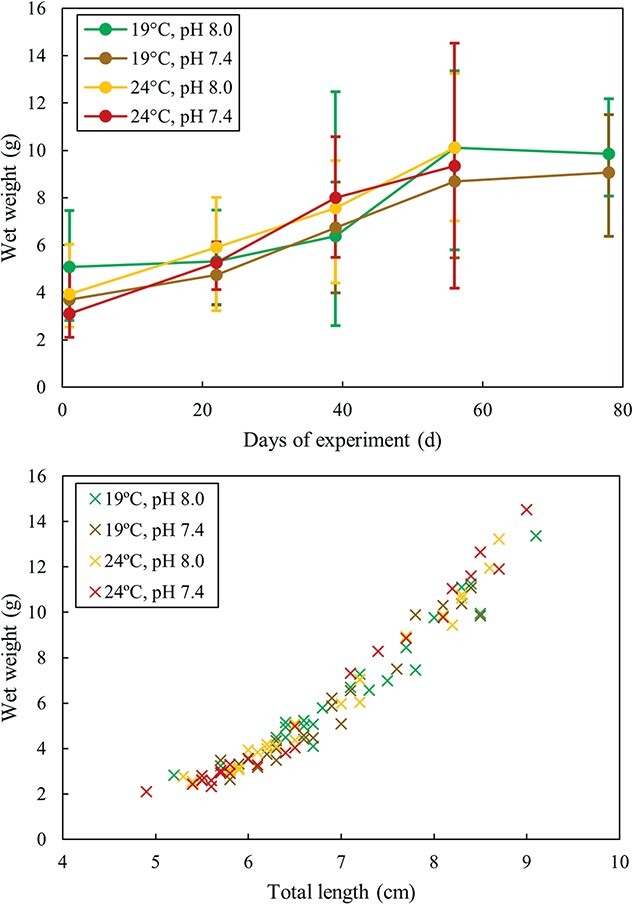
MARE-FCUL experimental data of wet weight versus time (top) and wet weight versus total length (bottom), for white seabream.

**Figure 5:**
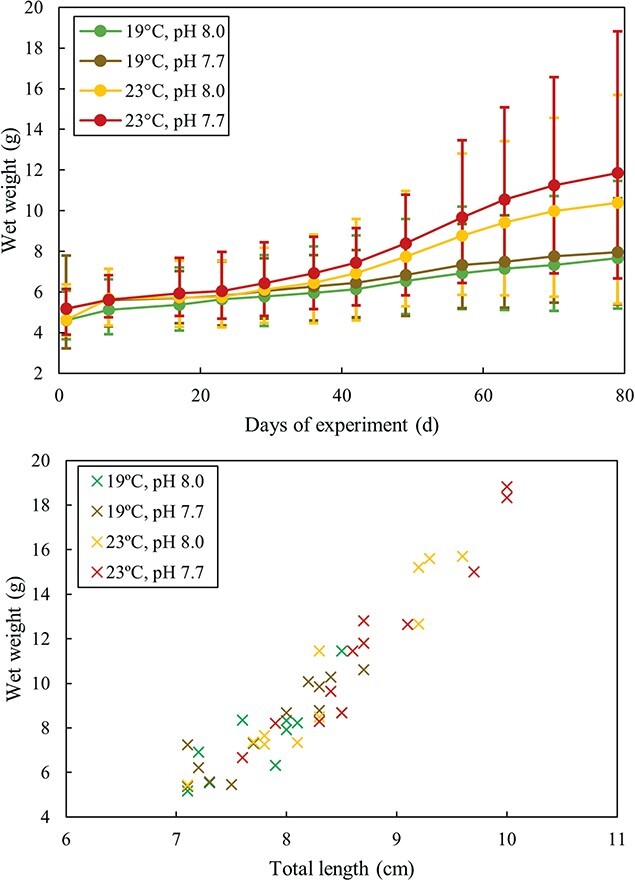
MARE-FCUL experimental data of wet weight versus time (top) and wet weight versus total length (bottom), for zebra seabream.

**Figure 6:**
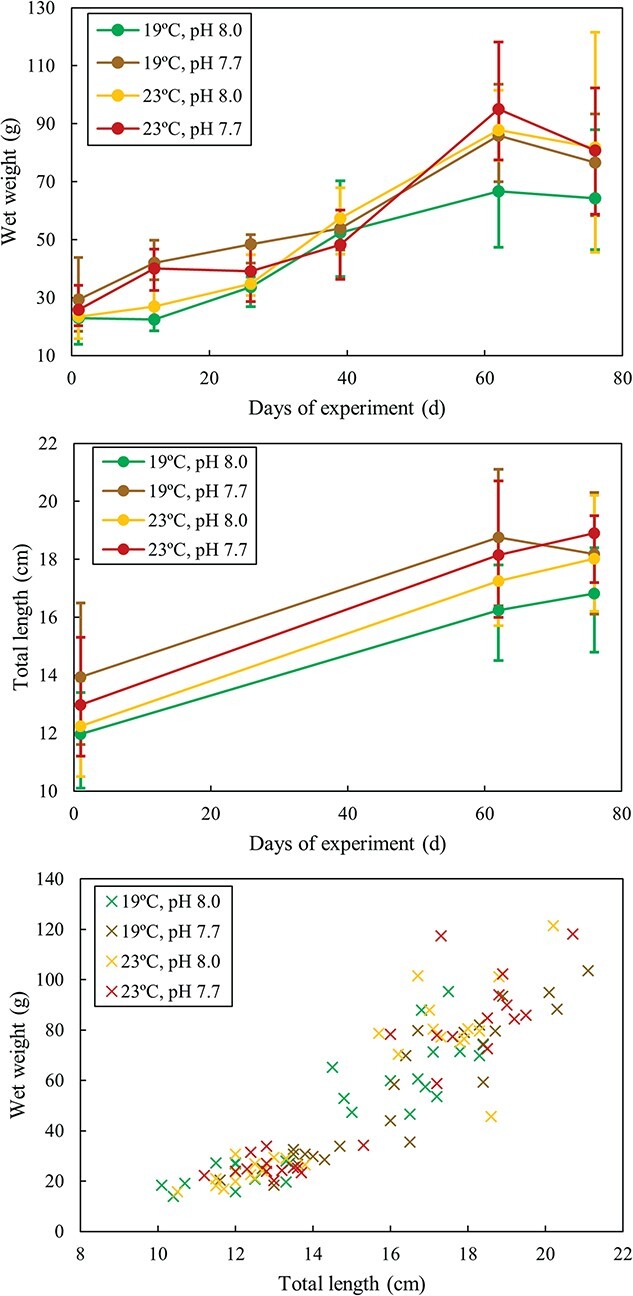
MARE-FCUL experimental data of wet weight versus time (top), total length versus time (middle) and wet weight versus total length (bottom), for Senegalese sole.

**Figure 7:**
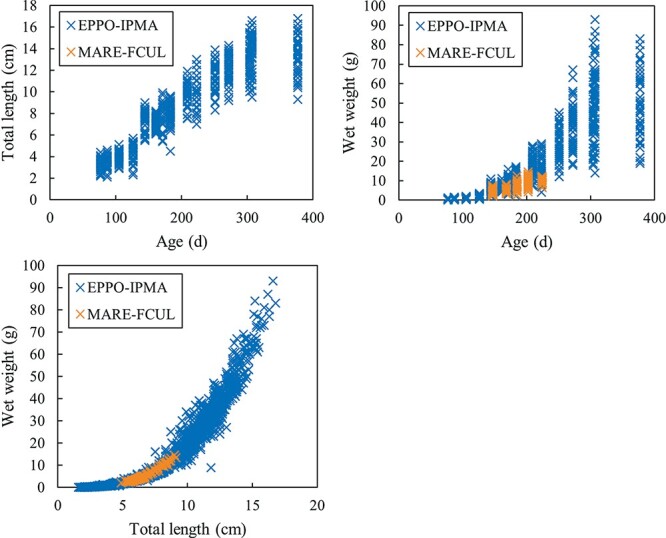
EPPO-IPMA experimental data of total length versus age (top-left), wet weight versus age (top-right) and wet weight versus total length (bottom-left), for white seabream, with MARE-FCUL data for comparison.

**Figure 8:**
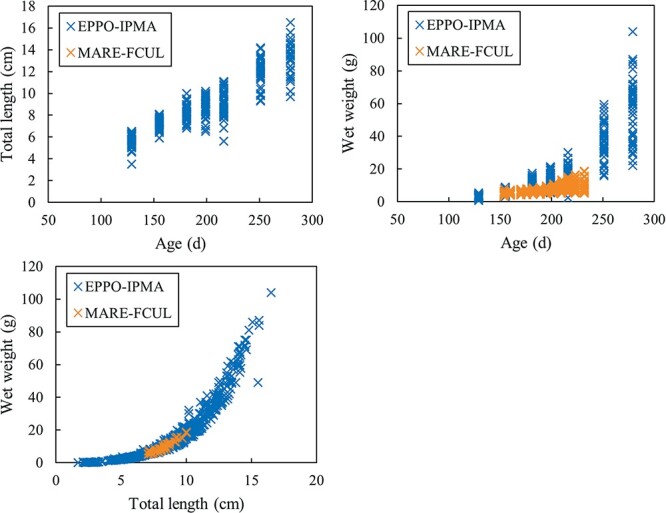
EPPO-IPMA experimental data of total length versus age (top-left), wet weight versus age (top-right) and wet weight versus total length (bottom-left), for zebra seabream, with MARE-FCUL data for comparison.

**Figure 9:**
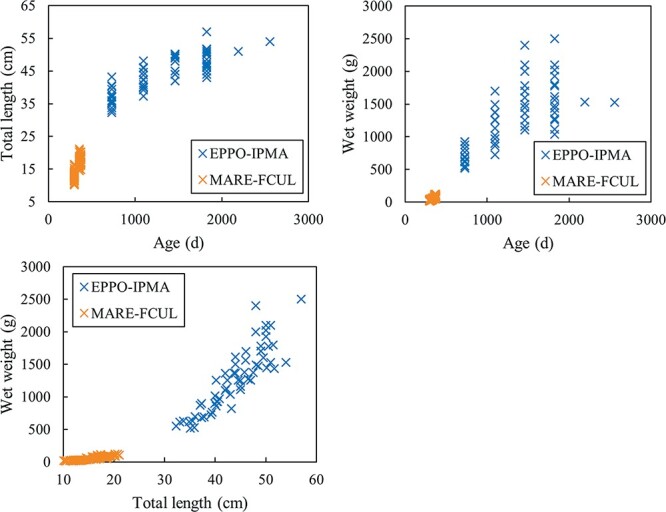
EPPO-IPMA experimental data of total length versus age (top-left), wet weight versus age (top-right) and wet weight versus total length (bottom-left), for Senegalese sole, with MARE-FCUL data for comparison.

## Appendix 1

Data from a large number of bibliographical references was included in the parameter estimation procedure, alongside the data from the experiments conducted for this study. Here, we present a summary of all zero-variate and univariate data used in the procedure.

**Table 5:** Zero-variate data used in the parameter estimation. Data that replaced the original data on the files from the AmP collection in bold. New data added to the files in italic

Data	*D. sargus*	*D. cervinus*	*S. senegalensis*
Age at hatching (d)	*3* ^[1]^	-	1.58 ^[9]^
Age at birth (d)	**7.5** ^[2]^	4 ^[*]^	3.58 ^[9]^
Age at metamorphosis (d)	*23* ^[3]^	-	19 ^[9]^
Age at puberty (d)	*776.8* ^[4]^	*1460* ^[6]^	1460 ^[10]^
Lifespan (d)	3650 ^[5]^	6205 ^[7]^	4015 ^[11]^
Egg diameter (cm)	-	-	0.1 ^[9]^
Length at hatching (cm)	*0.26* ^[5]^	-	0.1382 ^[9]^
Length at birth (cm)	-	-	0.2484 ^[9]^
Length at metamorphosis (cm)	-	-	0.9176 ^[9]^
Length at puberty (cm)	**16.73** ^[4]^	**27.3** ^[6]^	**30** ^[12]^
Ultimate length (cm)	*45* ^[5]^	**58.4** ^[6]^	**60** ^[12]^
Egg weight (g)	-	-	46.15 × 10^−6^ (dry) ^[9]^
Weight at hatching (g)	-	-	33.19 × 10^−6^ (dry) ^[9]^
Weight at birth (g)	*3.2 x 10* ^*−4* [5]^	3.2 × 10^−4 [8]^	35.4 × 10^−6^ (dry) ^[9]^
Weight at metamorphosis (g)	-	-	1.281 × 10^−3^ (dry) ^[9]^
Weight at puberty (g)	-	61.5 ^[7,8]^	-
Ultimate weight (g)	*1900* ^[5]^	**2700** ^[8]^	**1889** ^[*]^
Maximum reproduction rate (d^−1^)	*1468* ^[5]^	2085 ^[8]^	4160 ^[13]^
Reserve energy in the egg (J)	-	-	1 ^[9]^

References: ^[1]^Di Franco and Guidetti (2011), ^[2]^Ortiz-Delgado et al. (2003), ^[3]^Vigliola et al. (2000), ^[4]^Morato et al. (2003), ^[5]^FishBase (2021a), ^[6]^Pajuelo et al. (2003a), ^[7]^Pajuelo et al. (2003b), ^[8]^FishBase (2021b), ^[9]^Yúfera et al. (1999), ^[10]^Vinagre (2007), ^[11]^Teixeira and Cabral (2010), ^[12]^FishBase (2021c), ^[13]^Dinis et al. (1999), ^[*]^guess based on direct comparison with related species.

**Univariate data used in the parameter estimation:**

Time-length series for white seabream: Gordoa and Molí (1997).

Time-length series for zebra seabream: Pajuelo et al. (2003b).

Time-length series for Senegalese sole: Teixeira and Cabral (2010).

Time-weight series for Senegalese sole: Yúfera et al. (1999).

Time-length, time-weight and length-weight data for white seabream, zebra seabream and Senegalese sole: EPPO-IPMA and MARE-FCUL.

## Appendix 2

Experimental univariate data from EPPO-IPMA and MARE-FCUL was included in the parameter estimation procedure. This data is represented here, in a series of graphs.

MARE-FCUL experimental data for white seabream:

MARE-FCUL experimental data for zebra seabream:

MARE-FCUL experimental data for Senegalese sole:

EPPO-IPMA experimental data for white seabream, with MARE-FCUL data for comparison:

EPPO-IPMA experimental data for zebra seabream, with MARE-FCUL data for comparison:

EPPO-IPMA experimental data for Senegalese sole, with MARE-FCUL data for comparison:
